# Student perception of workplace-based assessment

**DOI:** 10.1111/tct.12057

**Published:** 2013-11-12

**Authors:** Alexander Nesbitt, Freya Baird, Benjamin Canning, Ann Griffin, Alison Sturrock

**Affiliations:** University College London Medical SchoolUK

## Abstract

**Background:**

Workplace-based assessment (WPBA) is key to medical education, providing a framework through which the trainee can be assessed and receive feedback in the clinical setting.

WPBA was introduced in 2008–2009 to students in year 4 at University College London Medical School (UCLMS). Students raised concerns about the lack of standardisation in grading. As a result, white-space areas were introduced on WPBA forms. The aim of this was to permit assessors to expand their feedback, thereby enhancing its developmental potential. The aim of the project was to assess student perception of WPBA at UCLMS, and to determine whether re-designing the form had altered this perception.

Students raised concerns about the lack of standardisation in grading

**Method:**

An online survey was circulated to students in year 4 at the end of the academic year 2009–2010, and was repeated with the next cohort of year–4 students at the end of the academic year 2010–2011. Students were asked to express a level of agreement with 12 statements and for free-text comments on their experience with WPBA. Survey responses were analysed using an unpaired two-tailed Student's *t*-test, and QSR NVivo was used to manage the thematic analysis of the free-text comments.

**Results:**

Although there was no significant difference in student perception between cohorts, the analysis of free-text comments highlighted several themes for discussion.

**Conclusion:**

Students at UCLMS find WPBA valuable in highlighting areas for improvement and obtaining personalised feedback. They find the grading of WPBA too subjective, and that the attitudes of the assessors sometimes reduce its educational value. Suggestions are made to improve the value of WPBA in undergraduate medical education.

**Figure 4:**
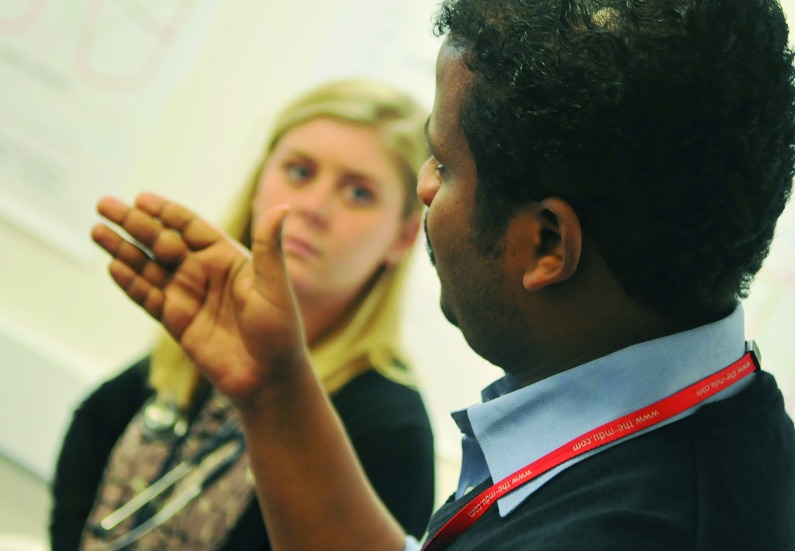

## INTRODUCTION

The lack of literature on WPBA in undergraduate medial education is problematic

Workplace-based assessment (WPBA) provides a framework through which trainees can be assessed and receive feedback in the workplace. It serves to assess a trainee's performance (what they ‘actually do’ in clinical practice), and therefore their readiness to advance.^1^ It provides an opportunity to identify trainees in need of additional support, and for developmental feedback to be given.^1^,^2^ WPBA has been adopted by the UK General Medical Council (GMC) and the Academy of Medical Royal Colleges (AoMRC) as a means of performance assessment in postgraduate medical education.^3^,^4^ It is also increasingly being used in undergraduate medical education.^3^

Feedback is an important influence on learner achievement,^5^ and medical students prize and welcome greater opportunities for constructive feedback.^6^ WPBA, if used correctly, represents an ideal opportunity to do this; however, despite subjective reports of the educational value of WPBA exercises, such as the mini-clinical evaluation exercise (mini-CEX) and case-based discussion (CBD), there is no evidence that they actually improve the performance of doctors and there has been no evaluation of their effect in undergraduate settings.^2^

Previous literature on trainees’ perception of WPBA produced mixed views. General Practitioner (GP) and Dermatology trainees valued the discussion of feedback and time spent face to face with their supervisor, but felt that the numerical grading system was unreliable, the time required for assessments was excessive and that they were difficult to schedule.^7^,^8^ Educational supervisors also highlighted this logistical issue.^9^ Supervisors described trainees as not being proactive enough with assessments, whereas trainees described assessors as not completing assessments with due care.^7^–^9^

The lack of literature on WPBA in undergraduate medical education is problematic. As students are not yet working, WPBA may perform differently than in the postgraduate environment. It is not known if medical students have different opinions of WPBA to postgraduates.

**Figure 3:**
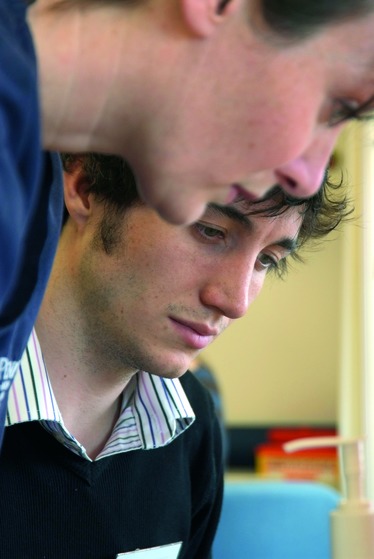


**Figure 1:**
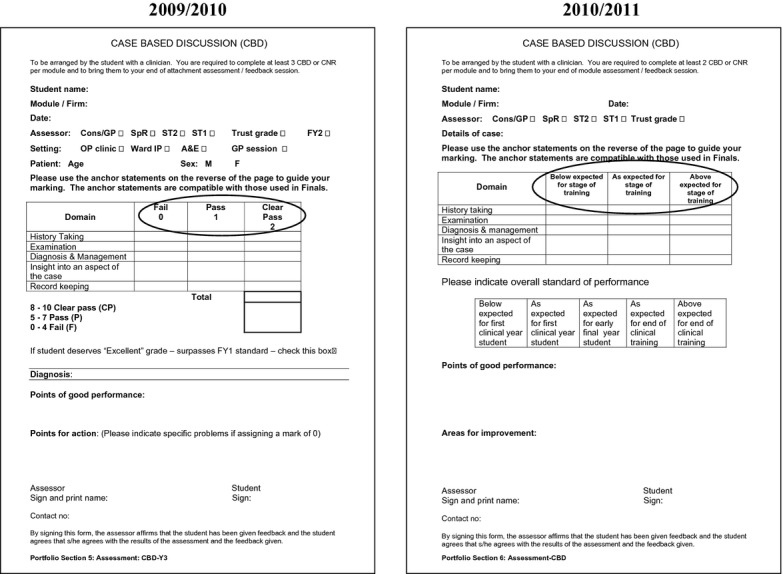
Images showing the change from grading to competency (from 2009–2010 to 2010–2011)

Since 2008, students at University College London Medical School (UCLMS) have completed a minimum of 16 WPBAs each year from year 4 (of a 6-year degree) onwards. This includes a combination of mini-CEXs, CBDs and case-note reviews (CNRs), which are an assessment of a student's written clerking. In 2008–2009, the scores received contributed to the end-of-year grade, but since 2009–2010, WPBAs have been used only in a formative manner. At the end of the 2009–2010 academic year a survey was sent to all students at the end of year 4 to gauge their perceptions of WPBA and identify any problems that they were having. Feedback from this survey led to a redesign of the forms, with scores being replaced by a description of the student's level of competence (Figure1). The survey was repeated with the next year-4 cohort to identify any change in perception.

This article reports the results of these surveys and focuses on the following questions.

- What is the student perception of WPBA at UCLMS?
- Has redesigning the WPBA forms altered students’ perception of WPBA?

## METHODS

An online survey was sent to year-4 students at the end of academic years 2009–2010 (*n* = 361) and 2010–2011 (*n* = 367). The survey was designed to collect both quantitative data (with a series of 12 statements and a five-point Likert scale to indicate the level of agreement) and free-text comments on students’ experience of WPBA.

An unpaired, two tailed Student's *t*-test was performed on responses to the statements, and free-text comments were analysed thematically. QSR NVivo 9 was used as the data management package and free-text data underwent a thematic analysis. The coding was independently reviewed by two other investigators until consensus was reached.

This study fell under UCL's exemption criteria for ethical approval, as it used survey data routinely collected from students.

## RESULTS

There were 170 responses (47%) from the 2009–2010 cohort and 118 responses (32%) from the 2010–2011 cohort. In total, 156 free-text comments were made, a sample of which appear in Table1 (2009–2010, 119; 2010–2011, 37). A number of themes emerged from the students’ responses to the survey, including subjectivity, purpose and educational value of assessments, as well as the attitude of the assessors.

Students felt their assessors often had negative views of WPBAs

**Table 1:** Free-text responses from students regarding their experience with workplace-based assessment (WPBA) at UCLMS

Free-text comments
Subjectivity of the assessment
‘I have found that for a similar performance the mark I am given might vary between 6 and 10, depending on the assessing doctor, for example.’ Survey 2009–2010
‘The main problem is the numerical marking. There is no consistency between doctors, some give all 10s, others refuse to give more than a 6. I think they should be changed so the only grades are fail, pass, clear pass.’ Survey 2009–2010
‘They are so variable with which doctor is filling them out for you. Some doctors only give an average mark for everyone, some always a top mark.’ Survey 2010–2011
Purpose of assessment (students believing WPBAs are summative)
‘I feel that the fact that the forms are subjectively marked out of 10 and then used for end-of-year quartiles needs to be changed.’ Survey 2009–2010
‘My main gripe with these assessments is the fact that they contribute towards the end-of-year assessment score.’ Survey 2009–2010
‘The marks are very dependent on individual doctor's subjectivity, so I think they would be more useful used as a tool to guide student's learning during the year rather than count towards an end-of-year score.’ Survey 2010–2011
Educational value of assessments
‘They are seen by most as a necessity to get done before the end of the module, stressing most students out, rather than being used as a learning tool.’ Survey 2009–2010
‘Many a time, the doctors just give any mark without giving any proper feedback or teaching.’ Survey 2009–2010
‘I always found the verbal/written feedback far more useful, as it specifically stated which aspects were good and which needed improvement.’ Survey 2009–2010
‘Work-based assessments are a useful exercise (when done properly) to get personalised feedback in examination and presenting skills.’ Survey 2009–2010
‘The problem with these assessments is that while good in theory, in practice they are extremely variable. Some of the feedback I have got from some doctors has been invaluable, but some of the feedback is not helpful at all. It can be very generic and mentions nothing specific to work on. For instance remarks such as “practise more” aren't very helpful. I assume all students plan to practise more anyway, but perhaps a particular area of weakness to practise on would be better.’ Survey 2010–2011
‘My personal tutor in XXX was fantastic as he made me come every week to his office and present to him to get a CBD. That was time put aside for forms and so feedback was great. I think this would generally be a good way for all students to get forms done.’ Survey 2010–2011
Attitude of assessor
‘The forms are seen, seemingly, as an annoyance – especially by more senior staff’ Survey 2009–2010
‘Some assessors seem to want to do the bare minimum, box ticking to get rid of the student, without even properly reading the form’ Survey 2010–2011
‘I have received numerous CEXs for examining patients even though I did not do so under supervision.’ Survey 2009–2010
‘Many people fabricate high marks, which is really frustrating to those of us who don't.’ Survey 2010–2011

Students were concerned about variability in marking, and that staff of different grades marked differently. Many comments from the 2009–2010 cohort expressed concerns that the marking system was not standardised, and that this was detrimental to the assessment. Students in this cohort also commonly expressed dismay that the assessments counted towards the end-of-year grade (which was inaccurate). Data from the survey suggest that students in both cohorts strongly disagreed or disagreed that the assessments should form part of the summative assessment (Table2).

Medical students fi nd WPBAs to be an effective mechanism for obtaining feedback

**Table 2:** Student responses to a selection of questions posed on workplace-based assessment (WPBA) in the online survey

	Cohort	Strongly disagree	Disagree	Neither agree or disagree	Agree	Strongly agree
The assessments are a useful way of making sure that supervising doctors spend time with me with patients and discussing cases	2009–2010	10.59%	25.88%	18.24%	33.53%	11.76%
	2010–2011	5.93%	30.51%	25.42%	32.20%	5.93%
I find the feedback from the assessments useful	2009–2010	11.18%	18.82%	22.35%	37.65%	10.00%
	2010–2011	7.63%	22.88%	25.42%	35.59%	8.47%
The assessments highlight things I would do differently in the future	2009–2010	6.47%	15.29%	26.47%	41.18%	10.59%
	2010–2011	4.24%	16.10%	32.20%	41.53%	5.93%
It is fair to use the assessments towards the end-of-year assessment score	2009–2010	62.35%	18.82%	5.29%	9.41%	4.12%
	2010–2011	33.90%	18.64%	17.80%	25.42%	4.24%
The assessments have been straightforward to organise	2009–2010	9.41%	42.35%	20.00%	24.12%	4.12%
	2010–2011	1.69%	27.97%	29.66%	37.29%	3.39%
The assessments interfere with the teaching time I have with the assessing doctor	2009–2010	4.71%	39.41%	25.29%	22.94%	7.65%
	2010–2011	2.54%	43.22%	29.66%	21.19%	3.39%

Comments regarding the educational value of the assessments were varied. Some students expressed the view that the assessments were a great opportunity to receive teaching and feedback, others that they were stressful and a nuisance. In both cohorts, students agreed or strongly agreed that feedback was useful, and that assessments highlighted things they would do differently in the future (Table2).

Students had mixed views on the quality of feedback they received, and suggested that assessments were most valuable when assigned a mentor, whose role was to aid in assessment and development (Table1).

The attitude of the assessors was highlighted in a negative manner by both cohorts, although students disagreed that assessments interfered with teaching time. Students had mixed views on whether assessments were useful in making supervising doctors spend time with them to discuss cases and examine patients (Table2).

It is important when examining the data in Table2 to note the proportion of students that appeared to give no opinion (‘neither agree nor disagree’). Additionally, the Student's *t*-test performed identified no significant difference between the survey responses of the two cohorts (p = 0.27–0.62).

## DISCUSSION

Both free-text and quantitative data from this study demonstrate that students at UCLMS find WPBA valuable in highlighting areas for improvement and obtaining personal feedback. In common with the postgraduate sector, medical students felt that WPBAs were useful for increasing contact time with seniors.^7^,^8^ Students also found WPBA to be most effective when allocated a tutor to complete assessments with, another finding supported by the literature.^10^ Students may see their tutor as a credible source of useful and accurate feedback as a result of a working relationship having been established.^10^

**Figure 2:**
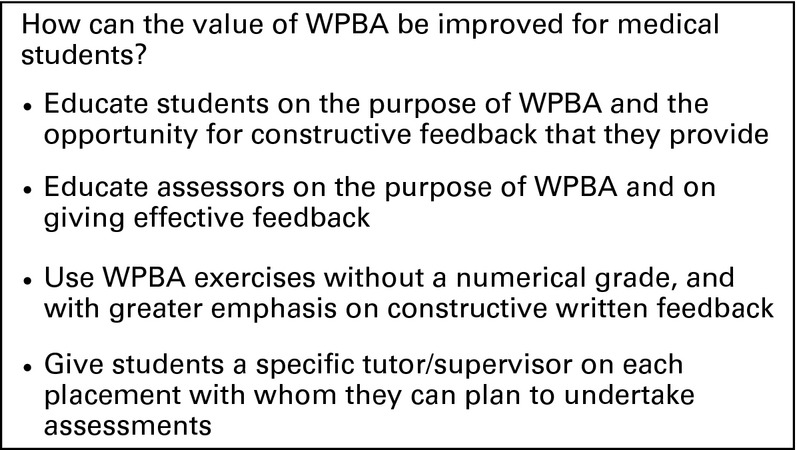
Changes to make in order to enhance the value of workplace-based assessment (WPBA) for medical students

Areas that students viewed with scepticism included the numerical grading system and inherent subjectivity of the assessment. Comments regarding the perceived unfairness of using WPBA as a summative assessment (Table1) may be a result of students misinterpreting the information provided by older peers. It was clear from the comments made that students felt their assessors often had negative views of WPBAs, viewing them as ‘tick-box’ exercises, and this had unhelpful repercussions on the students’ perceptions. These weaknesses have been previously reported and could be improved by better student and assessor education on WPBAs (Figure2).^7^,^8^

Despite the modifications made to WPBA forms between cohorts, there was no significant difference in the feedback. We believe this was because of students being unaware of the changes in the WPBA forms between the years.

From the data gathered in this study, it appears that medical student perception of WPBA is similar to that of postgraduate trainees, and the question is raised as to how that perception and educational value can be improved. Suggestions from our study and from the literature can be found in Figure2.

Limitations of the study include the low response rates for the surveys, the years being entirely separate cohorts and the involvement of only one medical school. The response rates may have been lower as the survey was distributed during the exam period; however, UCLMS is a large medical school, and the actual number of responses is substantial. The rates may have varied between years as a result of the first cohort having more of an impetus to comment in the light of their belief that WPBA was summative. Many students did not express a view, either negative or positive. We believe that this is a consequence of using a Likert scale that doesn't force an opinion. The recent introduction of WPBA for undergraduate students means that it may also have taken time for assessors to get used to them. Finally, using focus group data may have improved the strength of the conclusions drawn, as verbally expressed opinions may differ from those submitted online.

Despite its limitations, there are some valuable conclusions to be drawn from this study. Medical students find WPBAs to be an effective mechanism for obtaining feedback on performance, and that they are most effective when completed with an allocated tutor, but the negative attitudes of assessors impact upon their perceived value. This article adds a new perspective to the current literature on trainee perception of WPBA, and provides suggestions to improve WPBA at the undergraduate level (Figure2).

UCLMS is altering the WPBA forms so that more emphasis is put on formative free-text feedback than on grading, and is also piloting electronic WPBAs. Further research on the reception of these changes at UCL and other medical schools will add to the literature on WPBA in undergraduate medical education, both in terms of student perception and educational impact.
